# Supplementary material for: Binding-induced functional-domain motions in the Argonaute characterized by adaptive advanced sampling
Source: PLoS Comput Biol. 2021 Nov 29;17(11):e1009625. doi: 10.1371/journal.pcbi.1009625 (PMC8683029; doi:10.1371/journal.pcbi.1009625)
Supplement: S1 Fig — (PDF) [file pcbi.1009625.s001.pdf]

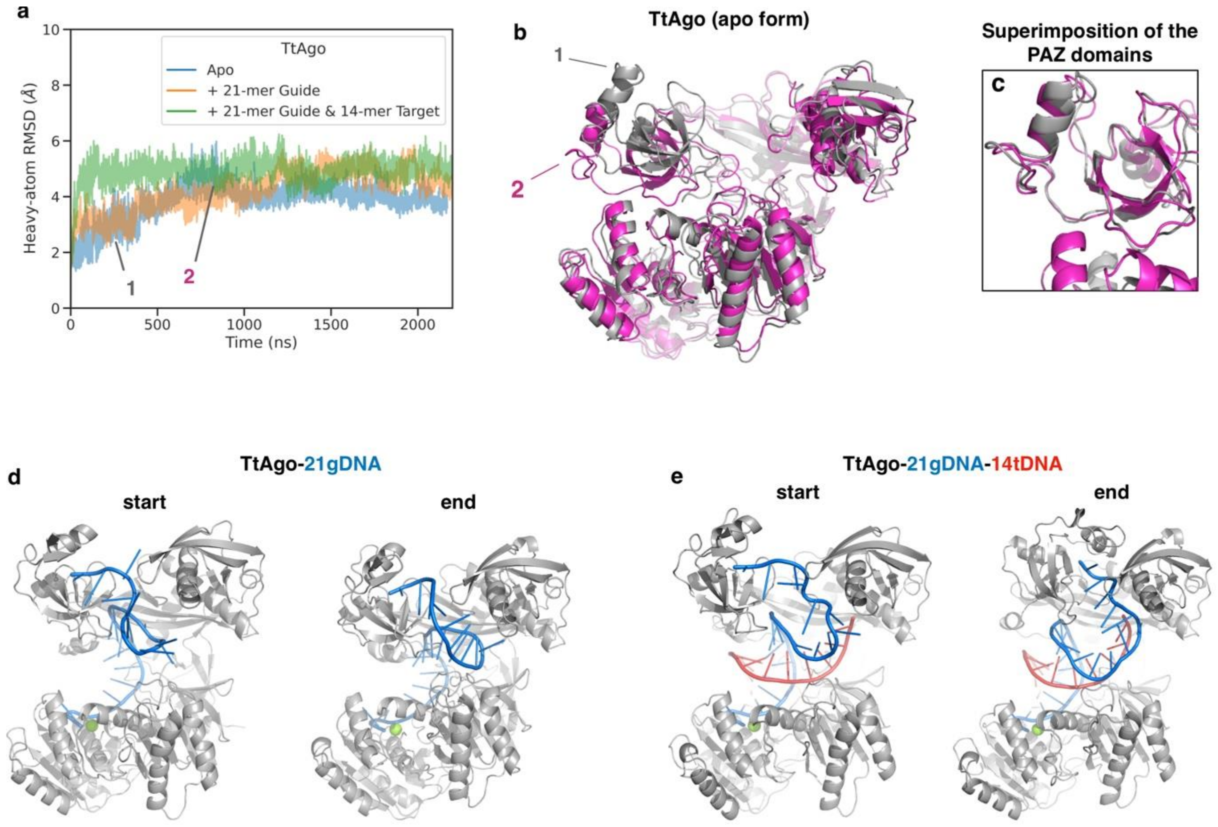

S1_Fig. (**a**) Heavy-atom RMSD versus time for three TtAgo structures; apo, binary (with 21-mer guide DNA) and ternary (with guide & 14-mer target DNA) structures. (**b**) Superimposition of two frames from the apo form simulation. (**c**) superimposition of the frames in **b** based on the PAZ domain amino-acid residues. The conformational changes within the PAZ domain are small (< 1.5 Å). Conformational snapshots of the binary and ternary complex simulations at the start and final simulation stages are shown in **d** and **e**, respectively.
