# Supplementary material for: Binding-induced functional-domain motions in the Argonaute characterized by adaptive advanced sampling
Source: PLoS Comput Biol. 2021 Nov 29;17(11):e1009625. doi: 10.1371/journal.pcbi.1009625 (PMC8683029; doi:10.1371/journal.pcbi.1009625)
Supplement: S2 Fig — (PDF) [file pcbi.1009625.s002.pdf]

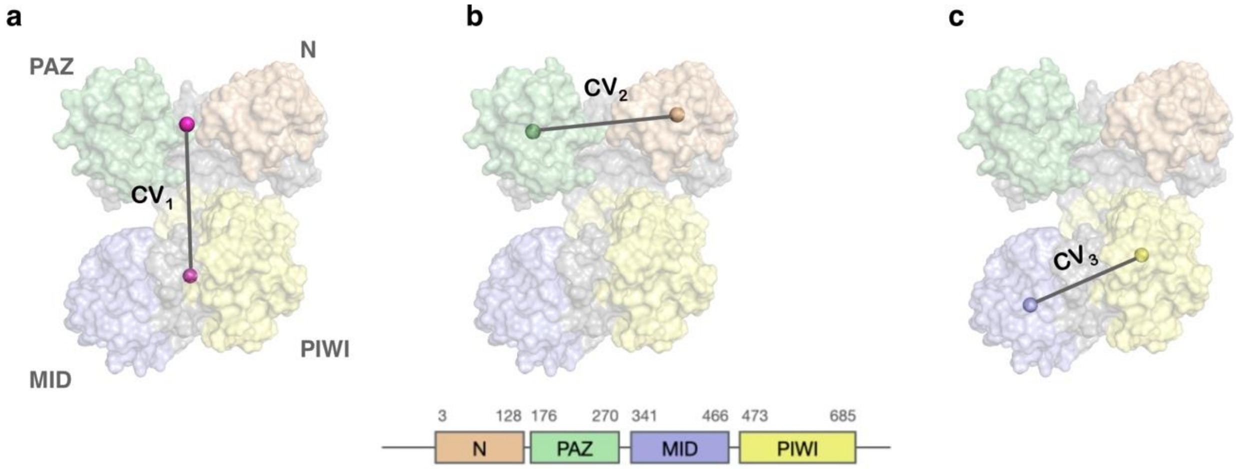

S2_Fig. Three center-of-mass (COM) distances served as collective variables. Each CV was independently biased by a potential to promote domain motions. The CVs were defined as the distance between centers-of mass of the two lobes (**a**), PAZ & N (**b**), and MID & PIWI (**c**).
