# Supplementary material for: Binding-induced functional-domain motions in the Argonaute characterized by adaptive advanced sampling
Source: PLoS Comput Biol. 2021 Nov 29;17(11):e1009625. doi: 10.1371/journal.pcbi.1009625 (PMC8683029; doi:10.1371/journal.pcbi.1009625)
Supplement: S3 Fig — (PDF) [file pcbi.1009625.s003.pdf]

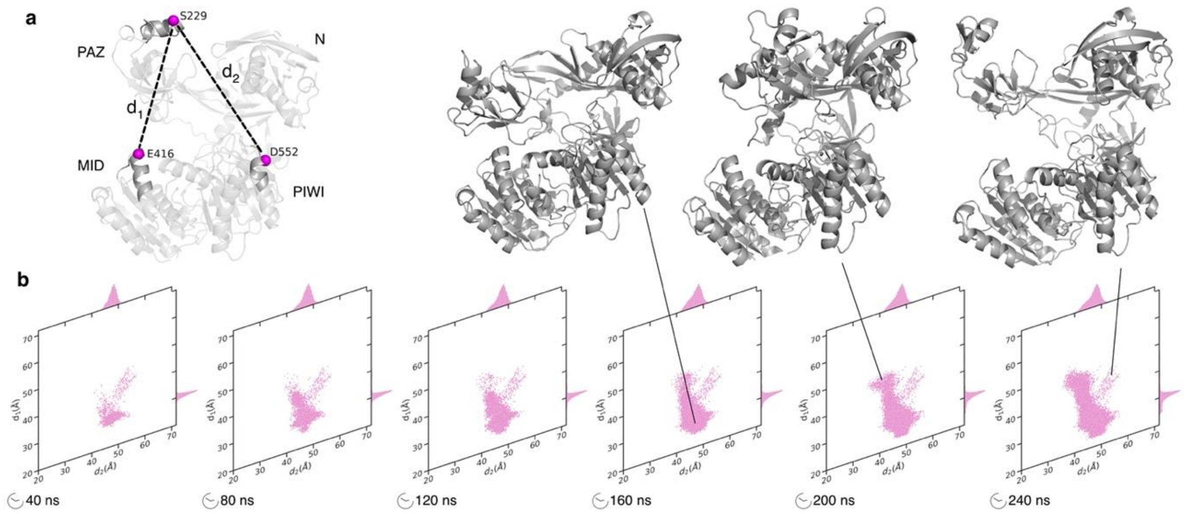

S3_Fig. Convergence of the H-REMD simulations were analyzed by monitoring PAZ conformational changes. PAZ position relative to MID and PIWI was measured by the α-Carbon distance between S229-E416 (d_1) and S229-D552 (d_2) respectively (**a**). **b**, plots show the distances d_1 vs. d_2 in 40 ns intervals. All data points are gathered from the reference replica.
