# Supplementary material for: Binding-induced functional-domain motions in the Argonaute characterized by adaptive advanced sampling
Source: PLoS Comput Biol. 2021 Nov 29;17(11):e1009625. doi: 10.1371/journal.pcbi.1009625 (PMC8683029; doi:10.1371/journal.pcbi.1009625)
Supplement: S4 Fig — (PDF) [file pcbi.1009625.s004.pdf]

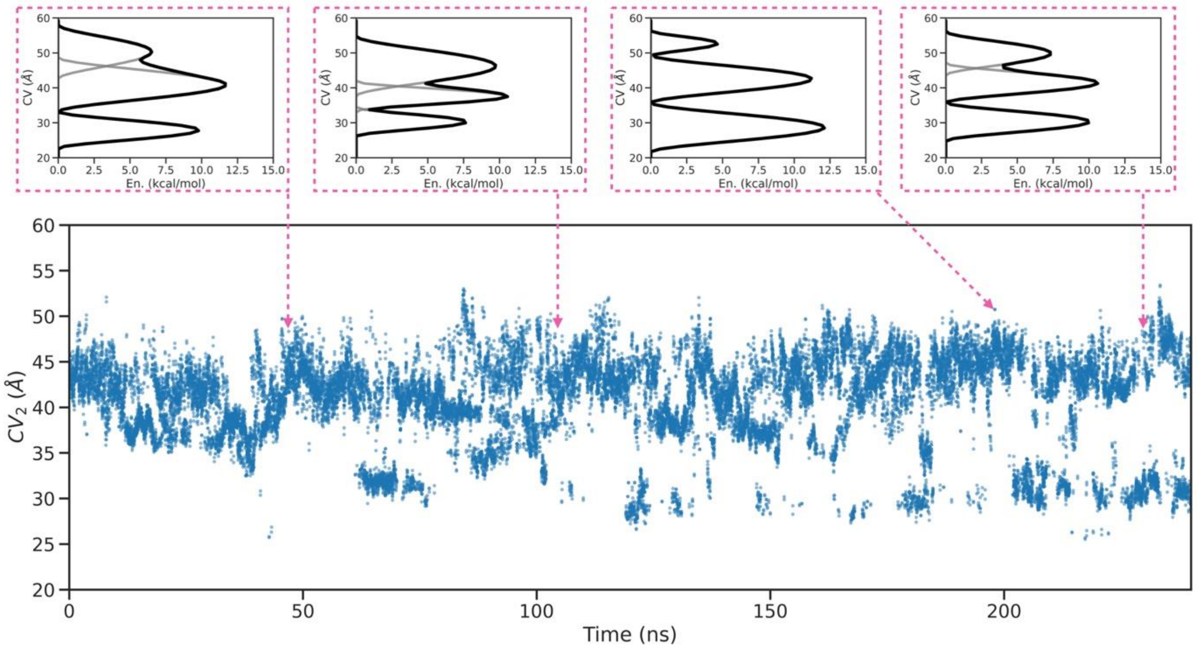

S4_Fig. The collective variable (CV) defined as the COM distance between PAZ and N domains (CV_2) as a function of time in the H-REMD simulation. The algorithm at every iteration evaluates the CV probability distribution and updates the bias potential accordingly. The sum of Gaussian components (grey lines) constructs the bias potential (black line) used in the H-REMD simulations linearly scaled in each replica (no biasing in the reference replica), shown here in four different stages.
