# Supplementary material for: Binding-induced functional-domain motions in the Argonaute characterized by adaptive advanced sampling
Source: PLoS Comput Biol. 2021 Nov 29;17(11):e1009625. doi: 10.1371/journal.pcbi.1009625 (PMC8683029; doi:10.1371/journal.pcbi.1009625)
Supplement: S5 Fig — (PDF) [file pcbi.1009625.s005.pdf]

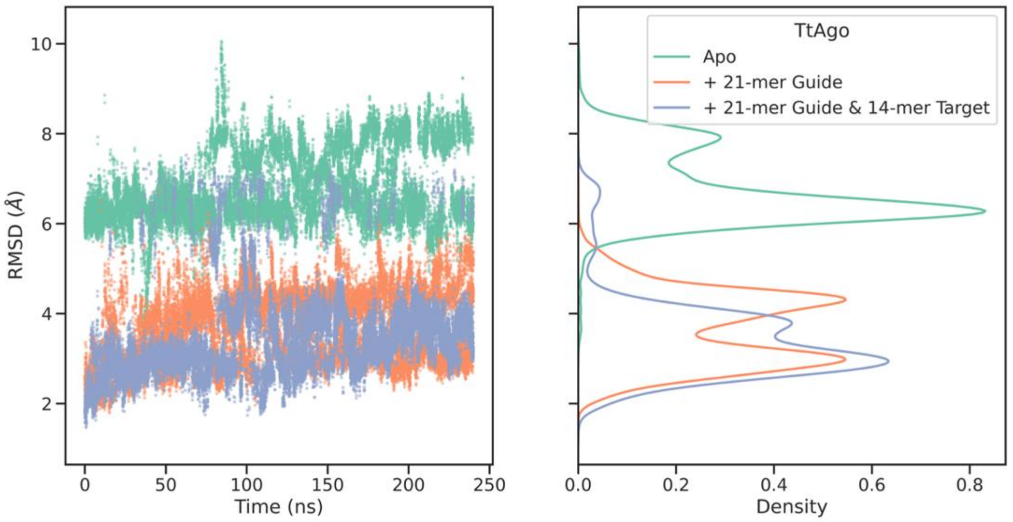

S5_Fig. RMSD plot vs. simulation time with respect to the initial structure (TtAgo:21-mer guide:14-mer target DNA-bound, PDB: 4n41) for apo Argonaute, binary and ternary complexes during 250 ns of H-REMD simulations (in the reference replica). The equilibration phase results were neglected. The probability distribution is plotted on the right.
