# Supplementary material for: Binding-induced functional-domain motions in the Argonaute characterized by adaptive advanced sampling
Source: PLoS Comput Biol. 2021 Nov 29;17(11):e1009625. doi: 10.1371/journal.pcbi.1009625 (PMC8683029; doi:10.1371/journal.pcbi.1009625)
Supplement: S6 Fig — (PDF) [file pcbi.1009625.s006.pdf]

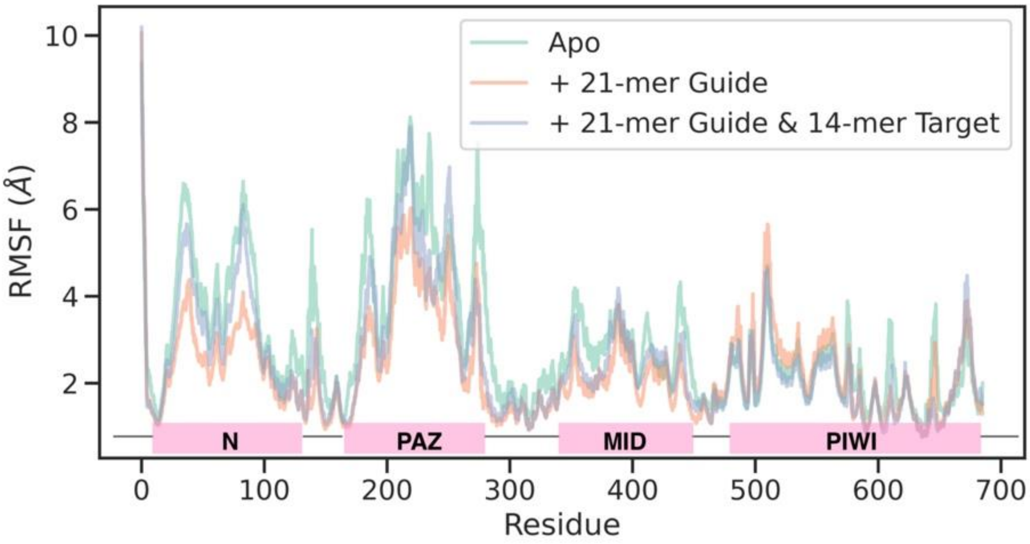

S6_Fig. RMSF of the Argonaute variants (apo, binary and ternary complex) obtained from the reference replica of the H-REMD simulations.
