# Supplementary material for: Binding-induced functional-domain motions in the Argonaute characterized by adaptive advanced sampling
Source: PLoS Comput Biol. 2021 Nov 29;17(11):e1009625. doi: 10.1371/journal.pcbi.1009625 (PMC8683029; doi:10.1371/journal.pcbi.1009625)
Supplement: S7 Fig — (PDF) [file pcbi.1009625.s007.pdf]

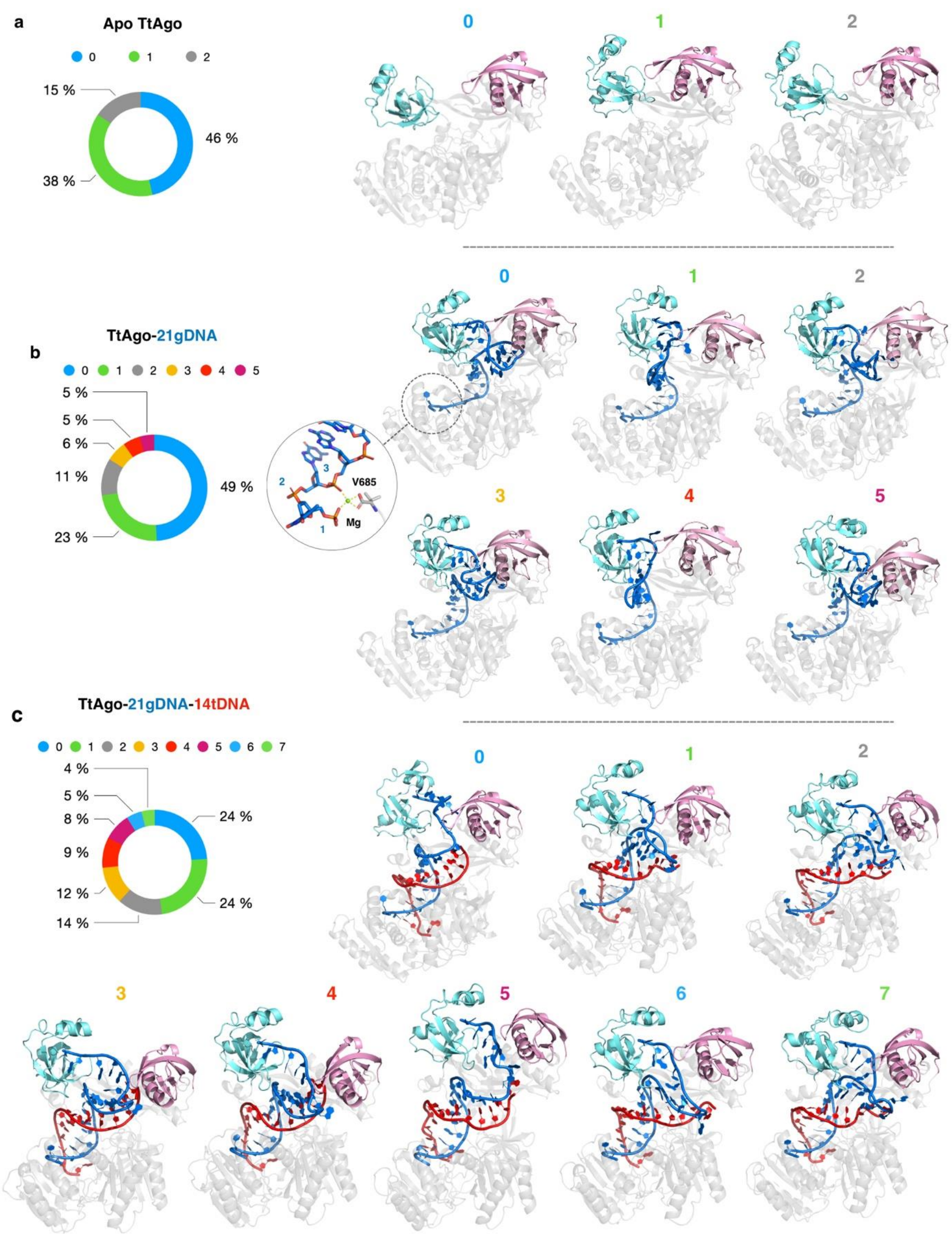

S7_Fig. Cluster populations and cluster representatives obtained from the H-REMD simulation trajectories of the apo form (**a**), binary (**b**) and ternary (**c**) complexes. The pie charts report on cluster populations. The PAZ and N domains are shown in cyan and pink cartoon respectively. The guide and target DNA strands are displayed in glue and red colors respectively.
