# Supplementary material for: Binding-induced functional-domain motions in the Argonaute characterized by adaptive advanced sampling
Source: PLoS Comput Biol. 2021 Nov 29;17(11):e1009625. doi: 10.1371/journal.pcbi.1009625 (PMC8683029; doi:10.1371/journal.pcbi.1009625)
Supplement: S8 Fig — (PDF) [file pcbi.1009625.s008.pdf]

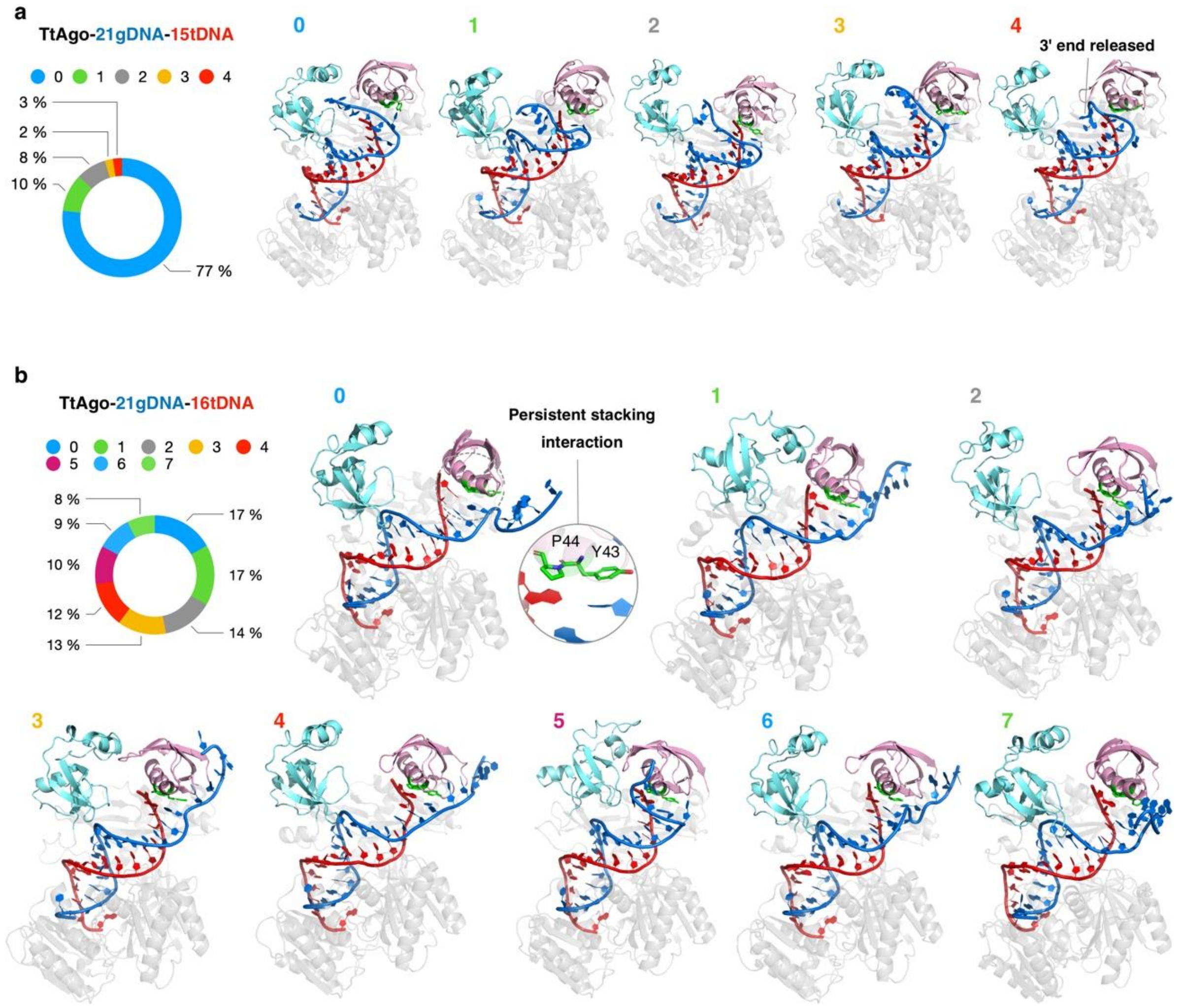

S8_Fig. (a) Cluster populations (pie charts) along with the cluster representatives of three guide/target-bound TtAgo proteins with varying target length: 15 (**a**) and 16 (**b**) nucleotide bases. The clustering was based on the 3' end - PAZ distance (OP2 phosphate atom of the 3' end and hydroxyl group of Y226).
