# Supplementary material for: Binding-induced functional-domain motions in the Argonaute characterized by adaptive advanced sampling
Source: PLoS Comput Biol. 2021 Nov 29;17(11):e1009625. doi: 10.1371/journal.pcbi.1009625 (PMC8683029; doi:10.1371/journal.pcbi.1009625)
Supplement: Attachment — Submitted filename: Response_DPD_MZ.pdf [file pcbi.1009625.s009.pdf]

Garching, 5.11.2021

Dear Dr. Skolnick, dear Dr. Elofsson

Thank you for returning our manuscript entitled “Binding induced functional domain motions in the argonaute characterized by adaptive advanced sampling" by D. Pourjafar-Dehkordi and M. Zacharias and the comments of the reviewers. In the following we like to comment on the concerns of the reviewers and indicate the changes and additions we have made to the manuscript. We also provide a version of the manuscript with all changes marked red.

Reviewer #1:
The study of how RNA is processed by cellular machineries is of central importance to nowadays chemical biology. Here Pourjafar-Dehkordi and Zacharias set out to study the recognition between guide RNA and mRNA target regions within the Argonaute protein. The authors carry out a comparative analyis of apo, guide and target bound systems. They use an adaptive sampling scheme based on H-REMD, convincingly showing relevant differences among the sampling in the different states (apo, binary, tertiary).
The paper is well conducted, and the differences in domain motions noticed are well described and based on solid calculations. I found this paper interesting to read and considering the growing importance of argonaute, it is worth having this paper exposed to the community. I think the paper is worth publishing as it is: it is very solid work, it contains a large amount of data, and everything is clearly presented.

Author reply: We thank the reviewer for the encouraging comment.

Reviewer #2:
The authors report molecular dynamics simulations of the important Argonaut protein, from which they obtain insights into domain motions, allostery, and other effects. An adaptive sampling method is introduced, which has similaritiies to metadynamics and allows enhanced sampling of slow domain motions. This is valuable as sampling domain motions is a hard and important problem, and the present method is not too complicated. The large and complex protein is simulated alone and with a series of its DNA or RNA substrates. The authors are able to explain its selectivity for nucleic acids above a certain length, which was not understood previously. The simulations and analysis are thorough and mostly well-explained. I have only a few comments on points of clarity.

1) The bias functions are changed every 10 ns; replica exchange then runs for another 10 ns, and so on. Can the authors say something about convergence of the statistical ensemble over this fairly short time segment? In particular, is it correct to say that the unbiased replica obeys its natural distribution of states, domain orientations, and so on, or is it continuously out of equilibrium 80 or 80% of the time?

Author reply: At the initial stage of the simulations the biasing potential used in the H-REMD scheme changes significantly. However, similar to meta-dynamics the biasing potential in each replica changes only little in advanced states of the simulation (demonstrated in Figure S4 in the SI). Hence the H-REMD simulation exchanges with replicas that with increasing simulation converge towards constant biasing potentials and then reach an equilibrium

distribution in each replica. Exchanges between the replicas according to the Metropolis acceptance criterion will not affect the equilibrium distribution in the reference replica (and all other replicas). However, even if the biasing potential is not completely converged the effect on the reference replica (without any biasing potential) will only be affected indirectly and moderately (through exchanges) but not directly as it is the case for meta-dynamics where only one simulation runs that includes the (changing) bias. This is also demonstrated already in the paper in Figure S3 that illustrates the convergence of the sampled domain distribution vs. simulation time for an example. We further discussed this issue on page 11 in the revised version.

2) In Fig 6, the distributions of states is converted into a free energy distribution, which describes the detachment of the nucleotide 3' terminus from the protein. Although the main result is clear (stability of the 14-mer), the free energis for the 15- and 16-mer rely on just a few detachment/reattachment events: around 10 for the 16-mer and just a handful for the 15-mer. Also, the 15-mer data are unclear, and seem to be hidden by the 14-mer points. Can the authors provide an error bar for the free energy curves and clarifiy the figure a bit?

Author reply: We agree with the reviewer that the Fig 6 needs improvement as suggested by the reviewer. We replaced Fig. 6 f/g by new plots indicating the sampling of the PAZ-3'-distance and a completely new plot of the calculated free energies for dissociating the 3'-end that also include error bars. The Figure legend was adapted accordingly.

Overall, there is a good level of novelty, a large and careful data set, and a number of interesting insights into the protein function. Experimental validation is mainly limited to the detachment of the longer nucleotide strands.

Reviewer #3:
The manuscript is an tour-de force description of a new approach to study Argonaute domain motion using simulations. It, unfortunately, is not very accessible to the non-expert. This is unfortunate, because the subject itself might be of interest to a wider audience (than biophysicists), too. The manuscript mentions "miRNAs" twice in the abstract and nowhere else after, which is strange and should be addressed by making clear how miRNAs are different from siRNAs and how AGO in non-miRNA organisms, such as Bacteria, act totally different from AGOs that load miRNAs and their targets (a discussion of target directed miRNA decay could be interesting).

Author reply: We agree with the reviewer that our explanation of the Argonaute key components was too brief and insufficient. We have modified and extended the description in the Introduction section. In the revised version the RISC complex, the miRNA and siRNA components of the AGO complex are explained and discussed and a comprehensive review on the system has been added to the reference list.

Finally, we like to thank the reviewers for the fair comments and hope that with the additions and changes we have made to the manuscript it is now acceptable for publication.

Yours sincerely,
Martin Zacharias
